# Health-related factors and their impact on blood-based biomarkers of Alzheimer's disease

**DOI:** 10.1016/j.nbas.2026.100166

**Published:** 2026-09-03

**Authors:** Carolin Kurz, Marleen Taute, Paulina Tegethoff, Anna Hufnagel, Sophia Leonhardt, Selim Üstün Gürsel, Carolin Koriath, Alexander Jethwa, Gwendlyn Kollmorgen, Tobias Bittner, Daniel Keeser, Matthias Brendel, Jan Haeckert, Julia Utecht, Boris Papazov, Johannes Levin, Günter Höglinger, Boris-Stephan Rauchmann, Robert Perneczky

**Affiliations:** a Department of Psychiatry and Psychotherapy, LMU Hospital, LMU Munich, 81377 Munich, Germany; b German Center for Neurodegenerative Diseases (DZNE), 81377 Munich, Germany; c Roche Diagnostics GmbH, 82377 Penzberg, Germany; d F. Hoffmann-La Roche Ltd, 4070 Basel, Switzerland; e Department of Nuclear Medicine, LMU Hospital, LMU Munich, 81377 Munich, Germany; f Munich Cluster for Systems Neurology (SyNergy), 80336 Munich, Germany; g Department of Psychiatry, Psychotherapy and Psychosomatics, Medical Faculty, University of Augsburg, 86156 Augsburg, Germany; h Department of Radiology, LMU Hospital, LMU Munich, 81377 Munich, Germany; i Department of Neurology, LMU Hospital, LMU Munich, 81377 Munich, Germany; j Department of Neuroradiology, LMU Hospital, LMU Munich, 81377 Munich, Germany; k Ageing Epidemiology (AGE) Research Unit, School of Public Health, Imperial College, London, UK; l Sheffield Institute for Translational Neuroscience, University of Sheffield, S10 2HQ Sheffield, UK; m Technical University of Munich, TUM School of Medicine and Health, Department of Psychiatry and Psychotherapy, TUM University Hospital, Munich, Germany

**Keywords:** Alzheimer's disease/blood, Biomarkers/blood, Apolipoproteins E/blood, Neurofilament proteins/blood, Tau proteins/blood

## Abstract

**Background:**

Health-related factors may influence blood-based biomarkers (BBBM) of Alzheimer's disease (AD). In this analysis, associations between modifiable factors and plasma biomarkers of neurodegeneration were investigated across the Alzheimer's disease spectrum and in cognitively healthy controls in a cerebrospinal fluid–confirmed (CSF) cohort.

**Methods:**

Plasma biomarkers included the Aβ1–42/1–40 ratio, pTau181, GFAP, NFL and ApoE4. Multiple linear regression was used to test associations with lifestyle factors (physical activity and sleep), physiological factors (including renal and lipid metabolism markers), genetic factors (APOE ε4), age and sex. Percentage effect sizes and confidence intervals were calculated.

**Results:**

The study included CSF-characterized individuals with AD (mild cognitive impairment due to AD and AD dementia) and cognitively healthy controls (*n* = 116; mean age 71.2 years). Overall, the associations were modest, with wide confidence intervals reflecting variability in the outcomes and the limited range of predictors in this relatively healthy sample. In CSF-confirmed participants, age emerged as the most consistent predictor of plasma biomarker levels, particularly NFL and pTau181. APOE ε3/ε4 genotype was additionally associated with higher pTau181 levels. Other demographic, metabolic and lifestyle-related variables showed only weak or inconsistent associations.

**Conclusion:**

To implement BBBM in broader populations, a systematic evaluation of confounders is required. As aging cohorts present with mixed pathologies, strategies to address heterogeneity will be essential. The limited number of robust associations observed suggests that plasma biomarkers are influenced primarily by age and genetic background rather than by metabolic factors in this cohort. Validation in more diverse populations remains warranted.

## 1 Introduction

As lifestyle factors contribute to the multifactorial etiology of sporadic Alzheimer's disease (AD), it remains unclear to what extent their biological signatures are reflected in blood-based biomarkers (BBBMs). [1] [2] [3] Recent population-based studies suggest that higher levels of physical activity and better sleep quality are linked to lower concentrations of plasma phosphorylated tau (pTau181), glial fibrillary acidic protein (GFAP), and neurofilament light chain (NFL), indicating beneficial effects on neuroinflammatory and neuroaxonal processes. [4] [5] [6] [7] [8] More recently, large memory-clinic and population-based studies have extended these observations by demonstrating associations between higher physical activity and lower plasma p-tau217 and NfL concentrations together with better cognitive performance, while emphasizing the potential of blood-based biomarkers for predicting incident dementia in community populations. [7] [9] In addition to their potential role in disease detection, BBBMs have also been proposed as tools to monitor the biological effects of modifiable risk factors and lifestyle interventions over time. [10] Furthermore, blood-based biomarkers are increasingly being investigated as pharmacodynamic and treatment-monitoring biomarkers to assess biological responses to disease-modifying therapies and lifestyle interventions, with emerging evidence supporting their potential while highlighting generally modest intervention-related effects. [11] However, findings on whether these associations are moderated by the apolipoprotein E (APOE) genotype remain inconsistent, with some studies reporting stronger effects in ε4 carriers, while others find no genotype interaction. [3] [4]

In addition to lifestyle, physiological factors such as renal function, lipid metabolism, and blood pressure can influence biomarker clearance and expression. [12] [13] Emerging evidence further indicates that body mass index and blood volume may substantially influence plasma biomarker concentrations through dilution effects, thereby affecting biomarker interpretation and amyloid PET classification, particularly in preclinical Alzheimer's disease. [14] Short-term biological fluctuations - for instance, circadian or postprandial changes - as well as habitual dietary patterns may further contribute to within-person variability in BBBMs, although evidence for dietary associations remains heterogeneous across biomarkers and requires further longitudinal validation. [11] [15] [16] Consequently, biomarker concentrations may reflect not only underlying neurodegenerative pathology but also individual biological and physiological characteristics. This complexity poses a challenge for the interpretation of BBBMs in clinical and research settings, especially since participants in AD trials are typically younger, healthier, more educated, and less ethnically diverse than the general patient population. [17] [18] [19] [20] [21] [22] [23] [24] [25] [26] [27] Such selection bias limits the generalizability of biomarker findings to real-world clinical contexts. As blood-based biomarkers move towards broader clinical implementation, understanding the impact of demographic, physiological, and lifestyle-related factors on biomarker variability becomes increasingly important. Despite increasing clinical interest in plasma biomarkers such as the amyloid-β1–42/1–40 ratio (Aβ1–42/1–40), pTau181, NFL, and GFAP, few studies have systematically examined how demographic, lifestyle, and physiological factors interact with these markers in well-characterized, biomarker-defined early-stage AD populations. [28] In particular, data from cohorts with cerebrospinal fluid (CSF)-supported diagnostic classification remain limited. The aim of this study was to investigate associations between modifiable and non-modifiable factors and key BBBMs in a combined cohort of individuals with MCI due to AD, AD dementia (ADD), and cognitively healthy controls. Rather than focusing on diagnostic performance, we aimed to characterize sources of biological variability in BBBMs and to identify factors that may influence their interpretation in clinical and research settings. The goal of this analysis was to clarify the biological context of BBBM variability and evaluate its implications for generalizability and translational value in real-world populations.

## 2 Methods and materials

### 2.1 Study design and participants

This observational cohort study investigated the associations between lifestyle and physiological factors, and blood-based biomarkers (BBBMs), in individuals with early Alzheimer's disease (AD). The dataset combined participants from the ActiGliA and AmyClear cohorts (ClinicalTrials.gov identifiers: NCT06224920 and NCT05059158), whose inclusion and exclusion criteria were identical. Both protocols were approved by the Ethics Committee of LMU Munich (project numbers 17–755, 17–569 and 18–606) and written informed consent was obtained in accordance with the Declaration of Helsinki.

### 2.2 Eligibility criteria

Participants were between 55 and 85 years of age, had at least 6 years of formal education, and were required to be free from major psychiatric, neurological, or unstable systemic illnesses. Alzheimer's disease dementia (ADD) and mild cognitive impairment due to AD (MCI-AD) were diagnosed according to the clinical criteria of the National Institute on Aging–Alzheimer's Association (NIA-AA, 2011). Biomarker classification was supported by cerebrospinal fluid (CSF) evidence of amyloid pathology (Aβ1–42/1–40 ratio < 0.055) and tau pathology (phosphorylated tau181 > 61 pg/mL), consistent with the A/T/N framework and aligned with the revised Alzheimer's Association diagnostic and staging criteria. [29] [30] [31] Both cut-off values represent validated in-house reference thresholds established by the Department of Laboratory Medicine at LMU University Hospital, Munich, based on internal quality control and previously published laboratory standards. [32] MCI-AD participants scored 24–30 on the MMSE and had CDR = 0.5, while ADD participants scored 18–24 on the MMSE and had CDR ≥ 1. Healthy controls (HC) were cognitively normal (MMSE ≥28, CDR = 0) and biomarker-negative, meeting the A−/T– profile based on Aβ1–42/1–40 ≥ 0.055 and phosphorylated tau181 ≤ 61 pg/mL. Participants with probable non-AD dementias (e.g., frontotemporal dementia, progressive supranuclear palsy, corticobasal syndrome, dementia with Lewy bodies), major depression, or significant cerebrovascular lesions on MRI were excluded. [33]

### 2.3 Rationale for group merging

As MCI-AD and ADD participants represent adjacent stages on the Alzheimer's disease (AD) continuum and due to the limited sample size within each subgroup, both groups were combined into a single ‘AD spectrum’ category for analyses 1–3. This maximizes statistical power and enables more robust estimation of associations between biomarkers and health-related variables. This approach is consistent with previous work examining shared pathophysiological markers across early and mild dementia stages. Associations between blood-based biomarkers and risk factors were then assessed across the combined cohort (AD spectrum + healthy controls (HC)) to capture the full range of variability in biological and lifestyle measures, and to evaluate their general influence independently of diagnostic status. All included participants underwent comprehensive neuropsychological assessment using the Consortium to Establish a Registry for Alzheimer's Disease (CERAD) neuropsychological battery, the Mini-Mental State Examination (MMSE), the Clinical Dementia Rating (CDR), and the Free and Cued Selective Reminding Test (FCSRT), as well as 3 T magnetic resonance imaging (Magnetom Skyra, Siemens) and blood and cerebrospinal fluid (CSF) biomarker assessments. [34] [35] [36] [37] Neuroimaging included 3-T magnetic resonance imaging (Magnetom Skyra, Siemens Healthineers, Erlangen, Germany) with standardized structural T1-weighted and fluid-attenuated inversion recovery (FLAIR) sequences for diagnostic characterization and assessment of cerebrovascular pathology. [38] [32] Blood sampling and sample handling were performed according to standardized operating procedures as previously described. [39] Plasma biomarkers (Aβ1–42, Aβ1–40, pTau181, ApoE4, NFL, GFAP) were quantified using the *Roche NeuroToolKit* on Roche Diagnostics assay platforms (Roche Diagnostics, Switzerland). In addition, plasma pTau217 was measured using the Fujirebio Lumipulse assay platform (Fujirebio, Japan). Cerebrospinal fluid (CSF) biomarkers were analyzed with immunoassays from *IBL International* (Gunma, Japan). All additional clinical laboratory variables were measured using standard reagents and assay platforms from Roche Diagnostics (Roche Diagnostics, Switzerland).

### 2.4 Lifestyle and physiological assessments

Physical activity and rest–activity cycles were objectively measured using the ActTrust 2 actigraphy device (Condor Instruments, São Paulo, Brazil), worn continuously for 14 days. Sleep quality was assessed using the Pittsburgh Sleep Quality Index (PSQI), and physical activity was additionally evaluated using the Physical Activity Scale for the Elderly (PASE). The PSQI is a widely used and validated measure of subjective sleep quality in older adults and has been applied in large aging and dementia-related cohorts. [40] PASE is a recommended self-report instrument for physical activity in older adults and has shown acceptable reliability and validity across multiple cultural adaptations. [41] Daytime sleepiness was measured with the Epworth Sleepiness Scale (ESS), which has demonstrated acceptable reliability and validity in older adults and in individuals with mild cognitive impairment or dementia. [42] [43] Symptoms suggestive of REM sleep behavior disorder were screened using the REM Sleep Behavior Disorder Screening Questionnaire (RBDSQ), a brief, validated tool with good diagnostic accuracy in patients with neurodegenerative diseases, including Parkinson's disease and dementia. [44] [45] Actigraphy provides objective estimates of sleep–wake patterns and circadian rest–activity rhythms and is considered reliable and feasible in older and cognitively impaired populations, including individuals with dementia. [46] [47] Alcohol consumption, body mass index (BMI), systolic and diastolic blood pressure, serum cholesterol and creatinine were recorded during clinical visits. Alcohol consumption was assessed as average self-reported daily intake and converted into weekly alcohol units according to UK standard unit definitions (1 unit = 8 g ethanol). Consumption exceeding 15 units per week was classified as harmful alcohol use. Body mass index (BMI) was calculated as weight divided by height squared (kg/m^2^). Blood pressure was measured during study visits using standard clinical procedures and systolic and diastolic values were recorded.

### 2.5 Statistical analysis

Descriptive statistics were calculated for all variables and stratified by diagnostic group. Continuous variables are reported as means and standard deviations (or medians and interquartile ranges where appropriate) and group comparisons were performed using Wilcoxon rank-sum tests for continuous data, and chi-squared or Fisher's exact tests for categorical data. To reduce dimensionality and minimize collinearity among correlated lifestyle and physiological variables, principal component analysis (PCA) with Varimax rotation was first performed on the following: questionnaire-based measures (PSQI, ESS, RBD-SQ and PASE); actigraphy-derived indices (relative activity and sleep efficiency); and clinical laboratory variables. PCA was used as an exploratory dimensionality-reduction approach to identify representative variables from clusters of correlated measures rather than to derive latent constructs for hypothesis testing. Component retention was guided by eigenvalues >1, scree plot inspection, and interpretability of the resulting components. Variables with the highest loadings on each retained component were selected as representative predictors for subsequent regression analyses. Representative component loadings were then used to select a subset of independent predictors for subsequent regression analyses. This data-driven approach ensured that the regression models incorporated the most informative and statistically stable dimensions of health-related behavior.

### 2.6 Primary analyses

Robust linear regression models (MM-estimation via lmrob) were conducted for log-transformed plasma biomarkers (amyloid-β1–42/1–40 ratio, phosphorylated tau181 [p-tau181], glial fibrillary acidic protein [GFAP] and neurofilament light chain [NFL]).

Based on the PCA results, representative variables from each domain were entered into the regression analyses, including age, sex, APOE ε4 status, LDL cholesterol, creatinine, PASE, PSQI, sleep efficiency, and relative amplitude. For transparency, full regression models are provided in the Supplementary Material. The primary regression models were prespecified based on clinical relevance and PCA-guided variable selection. In exploratory analyses, a predefined missingness-driven reduction procedure was applied when the number of complete observations was insufficient for stable model estimation. Multicollinearity was examined using variance inflation factors (VIF) and heteroskedasticity using the Breusch–Pagan test. Multivariate outliers were identified using Mahalanobis distance, missing data were handled using available-case analysis. For exploratory models including actigraphy-derived measures, the number of complete observations was evaluated separately for each biomarker outcome. If fewer than 20 complete observations were available, predictors with the highest missingness burden (relative amplitude, sleep efficiency, PSQI, PASE, LDL cholesterol) were sequentially removed according to a predefined hierarchy in order to preserve model stability while retaining the core covariates age, sex, APOE ε4 status, and renal function measures. Biomarker outcomes with fewer than 20 complete observations after this procedure were not analyzed further.

### 2.7 Secondary analyses

In participants with complete actigraphy data, separate regression models were computed that also included objective sleep parameters (e.g. total sleep time and number of awakenings), in order to explore their additional predictive value beyond that of subjective measures. These analyses were considered exploratory due to the smaller sample size of the actigraphy subgroup.

### 2.8 Correlation analyses

To assess the construct validity of subjective versus objective sleep and activity measures, partial Spearman correlations adjusted for age and sex were computed between actigraphy indices and questionnaire-based variables. This complementary step aimed to determine whether behavioral self-reports captured similar dimensions of variability to the actigraphy-derived metrics. To account for multiple testing, *p*-values were adjusted using the Benjamini-Hochberg false discovery rate (FDR) procedure within each family of related analyses. Both raw and FDR-adjusted *p*-values are reported. Percentage effect sizes with 95% confidence intervals were calculated to improve interpretability and visualized as forest plots. All analyses were conducted using RStudio version 2025.05.1 + 513 and IBM SPSS Statistics version 29. [48]

## 3 Results

**Fig. 1:**
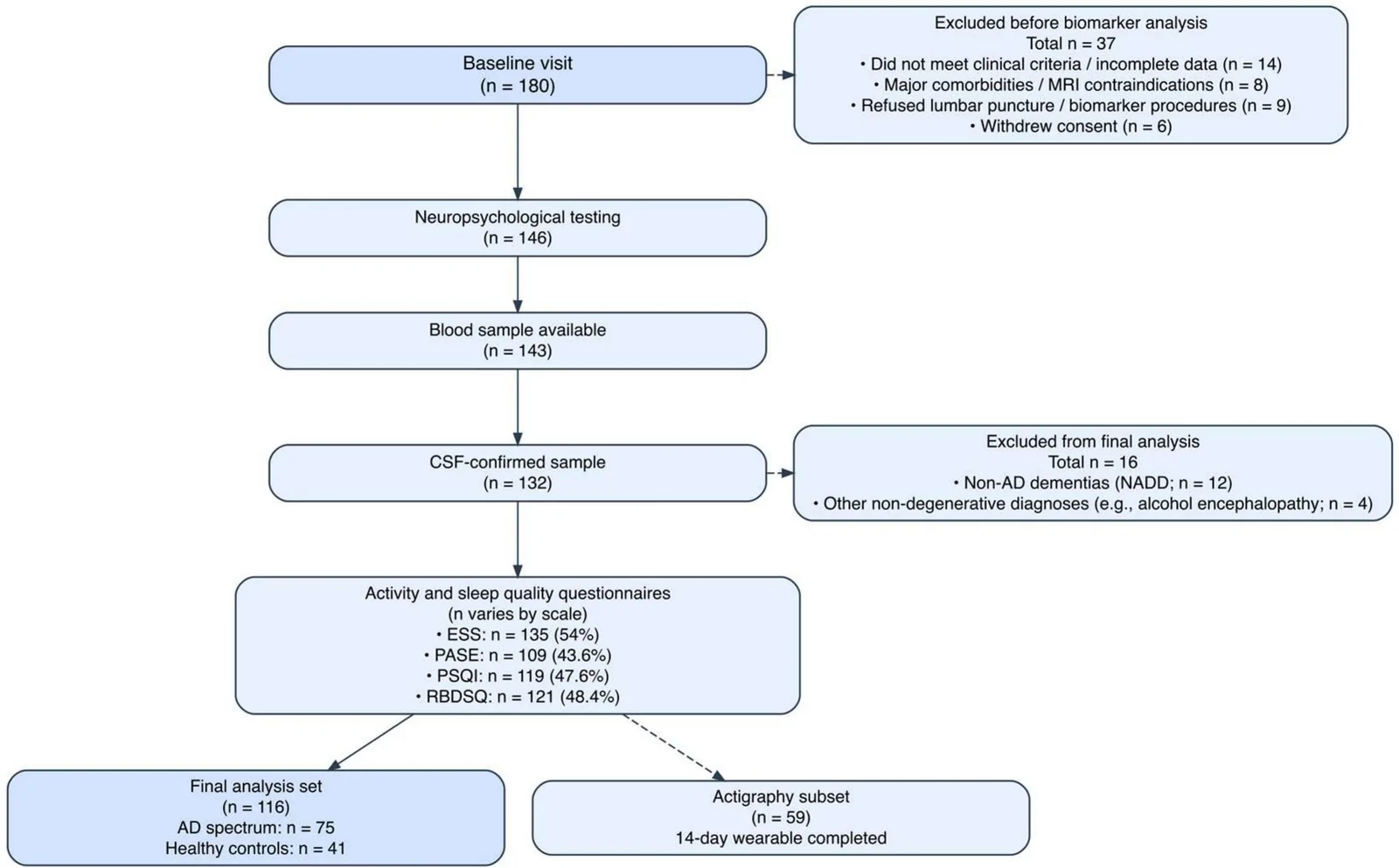
Flowchart recruitment of participants.

A total of 180 individuals were screened for eligibility. After exclusion of participants who did not meet study criteria, had incomplete datasets, relevant comorbidities, refused biomarker procedures, or withdrew consent, 143 individuals underwent CSF-supported diagnostic classification. Following exclusion of 16 participants with non-AD etiologies, the final analytical cohort comprised 116 participants, including 75 individuals within the AD spectrum (MCI-AD or AD dementia) and 41 cognitively healthy controls (Fig. 1). Actigraphy data were available for 59 participants. One multivariate outlier was excluded from regression analyses. The flow chart illustrates participant inclusion, exclusion, and data availability. A total of 180 individuals were assessed at baseline. Thirty-seven were excluded prior to biomarker analyses due to not meeting clinical criteria or incomplete datasets (*n* = 14), relevant comorbidities or MRI contraindications (*n* = 8), refusal of lumbar puncture or biomarker procedures (*n* = 9), or withdrawal of consent (*n* = 6). Among the 143 participants with cerebrospinal fluid (CSF)–confirmed diagnostic classification, 16 individuals were further excluded from the final analysis due to non-Alzheimer's disease etiologies (12 with non-AD dementias [NADD] and 4 with other non-degenerative diagnoses, e.g., alcohol encephalopathy). The final analytical cohort thus comprised 116 participants—75 within the Alzheimer's disease spectrum (mild cognitive impairment due to AD or AD dementia) and 41 cognitively healthy controls.

Of the total sample, neuropsychological testing data were available for 146 participants, blood samples for 143, CSF samples for 132, and actigraphy recordings for 59 participants. One multivariate outlier (healthy control) was excluded from the regression analyses based on Mahalanobis distance.

### 3.1 Descriptive data

The mean age of participants was 72 years (SD = 8) in the AD group and 68 years (SD = 8) in the HC group. This difference was statistically significant (*p* = 0.004). There was no significant difference in years of formal education between the two groups (AD: mean = 12.7, SD = 3.5; HC: mean = 13.5, SD = 2.9; *p* = 0.07). The mean body mass index (BMI) was similar in both groups (AD: 24.9 ± 3.9; HC: 26.2 ± 3.8; *p* = 0.11). Statistical power was limited and some non-significant findings (e.g. differences in BMI or education) may still be clinically relevant. Therefore, both statistical and potential clinical significance should be considered when interpreting these results. The sex distribution was balanced (AD: 48% male; HC: 57% male; *p* = 0.30) and there was no significant difference in household type (*p* = 0.20), with most participants living in a shared household. APOE ε4 carrier status was significantly more prevalent in the AD group (67%) than in the HC group (25%; *p* < 0.001). Missing data for individual variables ranged from 0.1% to 7% (see Table 1).

**Table 1:** Descriptive Statistics of Clinical and Demographic Variables by Group.

Characteristic	AD [*n* = 75 1]	HC [*n* = 41 1]	*p*-value 2
Age, years	72 (8)	68 (8)	0.004
(missing)	1	1	
Education, years	12.7 (3.5)	13.5 (2.9)	0.07
(missing)	11	10	
BMI, kg/m^2^	24.9 (3.9)	26.2 (3.8)	0.11
(missing)	25	21	
Sex			0.3
Male	38 (48%)	33 (57%)	
Female	41 (52%)	25 (43%)	
(missing)	2	4	
Household Type			0.2
Living alone	0 (0%)	0 (0%)	
With partner	15 (19%)	17 (30%)	
Shared household	55 (71%)	31 (54%)	
With relatives/children	4 (5.1%)	6 (11%)	
In assisted facility	4 (5.1%)	3 (5.3%)	
(missing)	3	5	
APOE4			<0.001
Non-carrier	25 (33%)	42 (75%)	
ε4 Heterozygous	50 (67%)	14 (25%)	
ε4 Homozygous	0 (0%)	0 (0%)	
(missing)	6	6	

Legend Table 1: Continuous variables are presented as mean ± standard deviation (SD), whereas categorical variables are presented as number (percentage). Age and education are reported in years, and body mass index (BMI) in kg/m^*2*^. Group comparisons were performed using independent-samples t-tests or Wilcoxon rank-sum tests for continuous variables, depending on data distribution, and Pearson's Chi-squared test or Fisher's exact test for categorical variables. A two-sided p-value < 0.05 was considered statistically significant.

1 n (%); Mean (SD)

2 Pearson's Chi-squared test; Wilcoxon rank sum test; Fisher's exact test

### 3.2 Neuropsychological performance, CSF biomarkers, and MRI measures

As expected, participants with CSF-confirmed Alzheimer's disease (AD) performed significantly worse across all neuropsychological measures than CSF-confirmed healthy controls (HC). Global disease severity, assessed by the CDR Sum of Boxes, was higher in the AD group (M = 2.56, SD = 2.08) than in HC (M = 0.04, SD = 0.14). Global cognitive function, as measured by the MMSE, was lower in the AD group (M = 25.28, SD = 3.46) than in the HC group (M = 29.46, SD = 0.98). The FCSRT revealed impaired episodic memory in AD, with lower Free Recall (M = 15.80, SD = 11.03) and Total Recall (M = 37.08, SD = 15.03) scores compared with HC (M = 29.73, SD = 5.46 and M = 47.73, SD = 4.60, respectively). Processing speed and executive function were also reduced in AD, with longer completion times on both the TMT-A and TMT—B. Likewise, the CERAD total score was significantly lower in AD, indicating more pronounced global cognitive impairment (Table 2). As expected based on the CSF-based diagnostic classification, participants with AD showed a significantly lower CSF Aβ42/40 ratio together with significantly higher CSF p-Tau181 and total tau concentrations than healthy controls (all *p* < 0.001), confirming the expected Alzheimer's disease biomarker profile (Table 2). Structural MRI measures showed the expected pattern of Alzheimer's disease-related atrophy, with significantly lower entorhinal cortex, temporal cortex, hippocampal, and posterior cingulate cortex volumes in the AD group, whereas estimated total intracranial volume (eTIV) was comparable between groups (Table 2).

**Table 2:** Descriptive statistics for neuropsychological performance and CSF biomarkers.

Variable	AD	HC	p
Neuropsychological measures			
CDR-SOB	2.56 (2.08)	0.04 (0.14)	<0.001
MMSE	25.28 (3.46)	29.46 (0.98)	<0.001
FCSRT Free Recall	15.8 (11.03)	29.73 (5.46)	<0.001
FCSRT Total Recall	37.08 (15.03)	47.73 (4.60)	0.002
TMT-A (s)	87.7 (44.91)	32.5 (10.56)	<0.001
TMT-B (s)	177.67 (90.36)	77.25 (21.63)	<0.001
CERAD Total Score	63.42 (17.40)	87.53 (4.05)	<0.001
CSF biomarkers			
Aβ42/40 ratio	0.037 (0.030–0.045)	0.083 (0.073–0.093)	<0.001
p-Tau181 (pg/mL)	91.4 (71.2–107.9)	52.1 (40.7–60.1)	<0.001
t-Tau (pg/mL)	446.1 (324.0–616.7)	222.0 (161.6–325.0)	<0.001
MRI measures			
Entorhinal cortex volume	2757 (649)	3561 (586)	<0.001
Temporal cortex volume	79,804 (12,366)	92,355 (11,115)	<0.001
Hippocampal volume	6843 (938)	8115 (1177)	<0.001
Posterior cingulate cortex volume	85,135 (11,938)	96,652 (12,398)	<0.001
eTIV	1,621,763 (167,163)	1,656,934 (180,339)	0.291

Legend Table 2: Neuropsychological performance, cerebrospinal fluid (CSF) biomarkers, and MRI measures in participants with Alzheimer's disease (AD) and healthy controls (HC). Continuous variables are presented as mean (SD) or median (IQR) for non-normally distributed variables (CSF biomarkers). Group differences were assessed using Welch's *t*-test or the Wilcoxon rank-sum test, as appropriate. Sample sizes varied across variables because not all participants completed all assessments. Abbreviations: CDR-SOB, Clinical Dementia Rating–Sum of Boxes; MMSE, Mini-Mental State Examination; FCSRT, Free and Cued Selective Reminding Test; TMT, Trail Making Test; CERAD, Consortium to Establish a Registry for Alzheimer's Disease; eTIV, estimated total intracranial volume.

### 3.3 Sleep and physical activity scores

In the actigraphy subsample (AD: *n* = 29, HC: *n* = 20), no significant group differences were observed for objectively measured sleep parameters, including sleep efficiency, wake after sleep onset, total sleep time, time in bed, number of awakenings, or circadian rhythm measures, with the exception of slightly higher interdaily stability in AD participants (*p* = 0.032; Table 3). Self-reported physical activity (PASE) did not differ between groups (*p* > 0.9). Participants within the AD spectrum reported better subjective sleep quality (PSQI: 3.8 ± 2.8 vs. 5.6 ± 3.6, *p* = 0.011) and lower daytime sleepiness (ESS: 5.0 ± 3.8 vs. 7.9 ± 4.0, *p* < 0.001) than cognitively healthy controls. No group differences were observed for RBDSQ scores (Table 3). To explore the relationship between subjective and objective sleep measures, partial correlations between actigraphy-derived parameters and sleep-activity questionnaires were examined (Supplementary Fig. S1). In addition, exploratory robust regression analyses were performed to assess associations between the two actigraphy-derived markers selected from the principal component analysis (sleep efficiency and relative amplitude; Supplementary Fig. S2) and plasma biomarkers (Supplementary Table S1). No association remained statistically significant after correction for multiple testing.

**Table 3:** Sleep and Physical Activity Scores and Questionnaires.

Actigraphy			
Characteristic	AD [n = 75 1]	HC [N = 41 1]	p-value 2
Recording time [days]	14.9 (4.5)	16.2 (5.5)	0.6
(missing)	52	42	
Sleep efficiency [%]	85.5 (6.8)	85.3 (5.2)	0.5
(missing)	52	42	
WASO [minutes]	57 (28)	60 (29)	0.7
(missing)	52	42	
Awakenings [n]	8.3 (3.4)	9.1 (3.5)	0.5
(missing)	53	42	
Total sleep time [hours]	6.26 (1.94)	6.27 (2.39)	0.5
(missing)	52	42	
Time in bed [hours]	7.29 (2.15)	7.34 (2.73)	0.5
(missing)	52	42	
Interdaily Stability	0.56 (0.11)	0.48 (0.12)	0.032
(missing)	52	42	
Intradaily Variability	3193 (5307)	3940 (5546)	0.7
(missing)	52	42	
Lowest 5 h activity	3561,577 (4,644,252)	2,508,195 (2,011,701)	0.3
(missing)	52	42	
most active 10 h activity	35,481,570 (10,424,231)	37,397,317 (13,522,552)	0.7
(missing)	52	42	
Relative Amplitude	0.83 (0.18)	0.86 (0.13)	0.3
(missing)	52	42	
**Questionnaires**			
Characteristic	AD [N = 75 1]	HC [N = 41 1]	p-value 2
PASE score [0–600]	367 (250)	353 (230)	>0.9
(missing)	28	28	
PSQI total [0−10]	3.8 (2.8)	5.6 (3.6)	0.011
(missing)	25	20	
ESS total [0–24]	5.0 (3.8)	7.9 (4.0)	<0.001
(missing)	21	13	
RBDSQ score [0−13]	7.1 (5.8)	8.0 (6.3)	>0.9
(missing)	29	22	

Legend Table 3: Actigraphy-derived sleep and circadian rhythm measures (available in a subset of participants) and questionnaire-based assessments. PASE = Physical Activity Scale for the Elderly; PSQI = Pittsburgh Sleep Quality Index; ESS = Epworth Sleepiness Scale; RBDSQ = REM Sleep Behavior Disorder Screening Questionnaire; WASO = Wake After Sleep Onset. Actigraphy variables included sleep efficiency, WASO, awakenings, total sleep time, time in bed, interdaily stability (IS), intradaily variability (IV), least active 5-h period (L5), most active 10-h period (M10), and relative amplitude (RA). Higher sleep efficiency and RA indicate better sleep continuity and circadian rhythm robustness, respectively.

1 Mean (SD)

2 Wilcoxon rank sum test, FDR-corrected

### 3.4 Health-related variables

Health-related variables were broadly comparable between groups (Table 4). Most laboratory parameters did not differ significantly between groups, including glucose, creatinine, liver enzymes, and lipid measures (Table 4). Smoking and alcohol consumption were generally low in both groups (Table S2).

**Table 4:** Health-related serum variables.

Characteristic	AD [n = 75 1]	HC [n = 41 1]	p-value 2
Glucose [mg/dl]	101 (24)	100 (38)	0.6
(missing)	15	16	
Creatinine (Jaffé method) [mg/dl]	0.88 (0.20)	0.90 (0.23)	0.6
(missing)	3	11	
GFR [ml/min/1.73m^2^]^3^	81.1 (14)	84.2 (13.9)	0.205
(missing)	5	10	
Aspartate Aminotransferase [U/l]	27 (10)	26 (8)	0.7
(missing)	15	16	
Alanine Aminotransferase [U/l]	19 (14)	20 (11)	0.6
(missing)	15	16	
Alcaline phosphatase [U/l]	74 (20)	75 (22)	0.7
(missing)	15	17	
Lactate dehydrogenase [U/l]	168 (43)	164 (37)	>0.9
(missing)	36	32	
Total cholesterol [mg/dl]	201 (47)	202 (37)	>0.9
(missing)	14	16	
Triglycerids [mg/dl]	120 (49)	124 (57)	>0.9
(missing)	17	19	
LDL cholesterol[mg/dl]	112 (36)	115 (32)	0.5
(missing)	18	19	

Legend Table 4: Descriptive statistics for routine serum laboratory parameters in participants with CSF-confirmed Alzheimer's disease (AD) and CSF-confirmed healthy controls (HC). Data are presented as mean (SD). Group comparisons were performed using the Wilcoxon rank-sum test with Benjamini–Hochberg false discovery rate (FDR) correction. Missing values are reported for each variable. eGFR was calculated using the CKD-EPI equation.

1 Mean (SD)

2 Wilcoxon rank sum test, FDR-corrected

### 3.5 Blood-based biomarkers

**Fig. 2:**
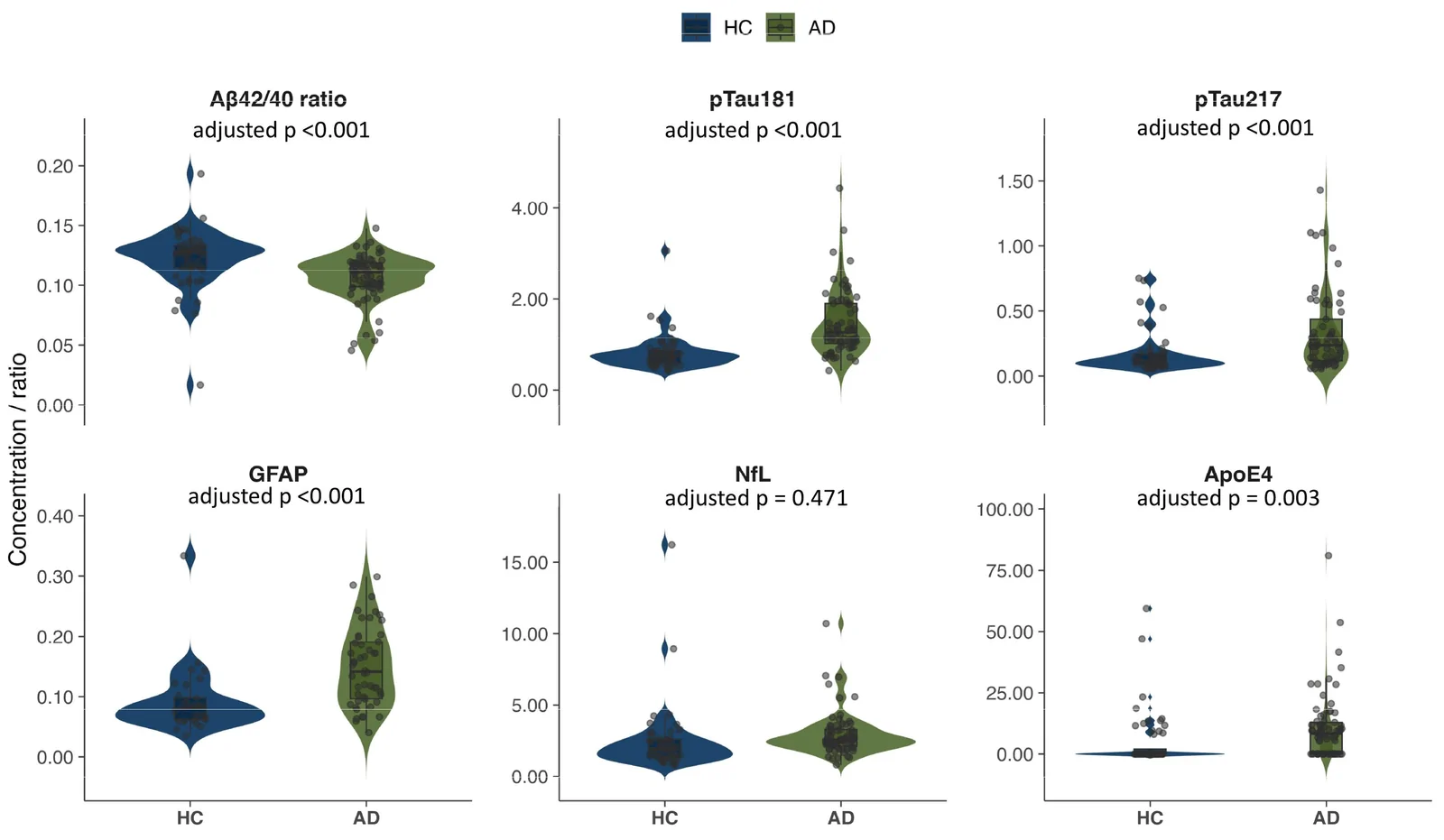
Group differences in standardized plasma biomarker concentrations.

Plasma biomarker levels showed significant differences between diagnostic groups, particularly for the Aβ1–42/1–40 ratio, ApoE4, NfL, pTau181, and pTau217 concentrations. The Aβ1–42/1–40 ratio was markedly lower in the AD group (M = 0.107, SD = 0.020) compared with healthy controls (M = 0.123, SD = 0.024; p < 0.001). Likewise, ApoE4 levels were significantly higher in AD (M = 11 mg/mL, SD = 14) than in HC (M = 5 mg/mL, SD = 11; p = 0.003). Although mean GFAP concentrations were higher in AD (M = 0.15 ng/mL, SD = 0.06) than in HC (M = 0.09 ng/mL, SD = 0.05), this difference was not statistically significant after correction for multiple testing (p = 0.471). NfL levels were significantly higher in AD (M = 2.93 pg/mL, SD = 1.55) compared with HC (M = 2.41 pg/mL, SD = 2.31; p < 0.001). Similarly, pTau181 concentrations were significantly elevated in the AD group (M = 1.45 pg/mL, SD = 0.71) relative to controls (M = 0.81 pg/mL, SD = 0.40; p < 0.001). pTau217 concentrations were likewise significantly higher in participants with AD (M = 0.35 pg/mL, SD = 0.29) than in healthy controls (M = 0.18 pg/mL, SD = 0.16; p < 0.001). No significant group differences were found for Aβ1–42 (p = 0.20) or Aβ1–40 (p = 0.70). Substantial overlap between groups was observed for all biomarkers despite significant group-level differences (Table 5 and Fig. 2).

**Table 5:** Descriptive statistics for plasma biomarkers.

Characteristic	AD [n = 75 ^1^]	HC [n = 41 1]	p-value 2
Aß1–42 [pg/ml]	24 (10)	26 (10)	0.2
(missing)	9	5	
Aß1–40 [pg/ml]	223 (66)	216 (70)	0.7
(missing)	11	6	
Aß1–42/1–40 ratio	0.107 (0.020)	0.123 (0.024)	<0.001
(missing)	11	6	
Apoe4 [mg/ml]	11 (14)	5 (11)	0.003
(missing)	9	5	
Gfap [ng/ml]	0.15 (0.06)	0.09 (0.05)	0.471
(missing)	32	30	
NFL [pg/ml]	2.93 (1.55)	2.41 (2.31)	<0.001
(missing)	11	9	
pTau181 [pg/ml]	1.45 (0.71)	0.81 (0.40)	<0.001
(missing)	11	8	
pTau217 [pg/ml]	0.35 (0.29)	0.18 (0.16)	< 0.001
(missing)	18	17	

Legend Table 5: Descriptive statistics for plasma biomarkers in participants with CSF-confirmed Alzheimer's disease (AD) and CSF-confirmed healthy controls (HC). Data are presented as mean (SD). Group comparisons were performed using the Wilcoxon rank-sum test with Benjamini–Hochberg false discovery rate (FDR) correction. Missing values are reported for each biomarker.

1 Mean (SD)

2 Wilcoxon rank sum test, FDR-corrected

While the raincloud plots illustrate statistically significant mean differences between AD and HC for the Aβ1–42/1–40 ratio, ApoE4, NfL, pTau181, and pTau217 (ApoE4: *p* = 0.003; all others *p* < 0.001; Fig. 2 and Table 5), substantial overlap between diagnostic groups remained evident for all biomarkers. The clearest separation was observed for pTau181 and pTau217, whereas the Aβ1–42/1–40 ratio and ApoE4 showed considerable within-group variability. Although mean GFAP concentrations were higher in AD, distributions overlapped extensively and the group difference was not statistically significant after correction for multiple testing (*p* = 0.471).

### 3.6 Regression analyses

**Fig. 3:**
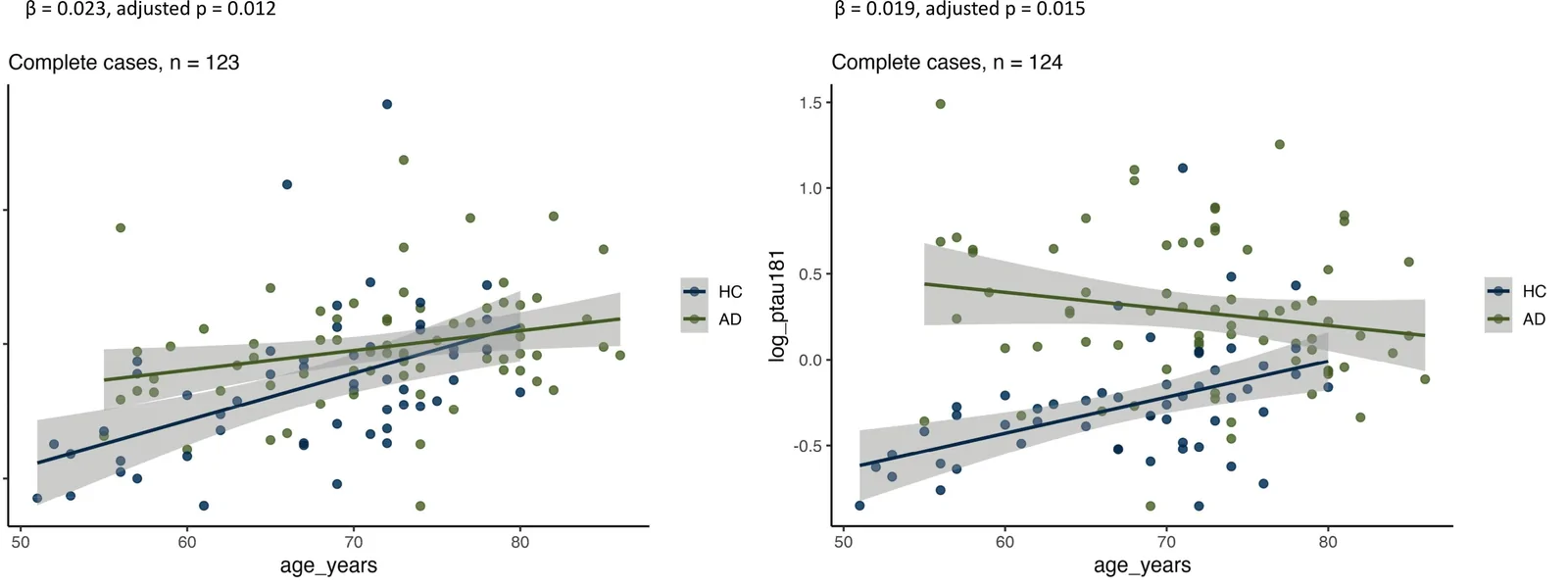
Association between age and plasma NfL and pTau181 levels. Scatterplots showing the association between age and log-transformed plasma neurofilament light chain (NfL; left) and phosphorylated tau 181 (pTau181; right) concentrations. Each point represents one participant. Solid lines indicate linear regression fits with 95% confidence intervals. Participants with Alzheimer's disease are shown in green and cognitively healthy controls in blue. Analyses were restricted to complete cases. Regression lines provide univariable visualization of the age association, whereas β coefficients and FDR-adjusted p-values correspond to the fully adjusted robust regression models presented in Table 6.

Robust regression analyses were performed in CSF-confirmed participants to identify demographic, genetic, metabolic, and lifestyle-related factors associated with plasma biomarker concentrations (Table 6). Age emerged as one of the most consistent predictors across models. Higher age was associated with increased plasma NfL concentrations in both amyloid-positive and amyloid-negative participants and with higher pTau181 and GFAP levels in selected subgroup analyses. APOE ε4 genotype was strongly associated with biomarker variability, including lower Aβ1–42/1–40 ratios and markedly higher plasma ApoE4 concentrations. In amyloid-positive participants, APOE ε3/ε4 carrier status was additionally associated with higher plasma pTau181 levels. Among physiological variables, higher serum creatinine concentrations were associated with increased plasma pTau181 (β = 0.715, *p* = 0.007), GFAP (β = 0.679, *p* = 0.026), and NfL (β = 0.536, *p* = 0.034) levels in sensitivity analyses. Because creatinine reflects multiple physiological processes, including renal clearance and body composition, these findings should be interpreted cautiously. Lifestyle-related variables showed comparatively limited associations. Poorer subjective sleep quality (higher PSQI scores) was associated with higher NfL concentrations in amyloid-negative participants (β = 0.075, *p* = 0.010). Physical activity (PASE) showed a modest inverse association with plasma ApoE4 concentrations, whereas no consistent associations were observed across the remaining plasma biomarkers. Overall, effect sizes were generally modest and confidence intervals were wide, reflecting substantial biological variability within this relatively healthy cohort. Fig. 3 summarizes the percentage effect sizes and corresponding confidence intervals for significant associations.

**Table 6:** Robust regression analyses of plasma biomarkers in CSF-confirmed participants.

Biomarker	Model	Predictor	β (95% CI)	Raw p	FDR-adjusted p
NfL (log)	Main (eGFR)	Age	0.023 (0.010–0.036)	<0.001	0.012
	Sensitivity (creatinine)	Age	0.025 (0.014–0.036)	<0.001	<0.001
pTau181 (log)	Sensitivity (creatinine)	Age	0.019 (0.008–0.031)	0.001	0.015
		APOE ε3/ε4 genotype	0.461 (0.141–0.782)	0.006	0.050

Legend Table 6: Results of multivariable robust linear regression analyses (MM-estimation, lmrob) for log-transformed plasma biomarkers in CSF-confirmed participants. Only associations remaining significant after Benjamini–Hochberg false discovery rate (FDR) correction are shown. The main analyses included eGFR as a marker of renal function, whereas sensitivity analyses replaced eGFR with serum creatinine. Regression coefficients (β), 95% confidence intervals (CIs), raw *p*-values, and FDR-adjusted *p*-values are reported.

## 4 Discussion

### 4.1 Key findings and implications

This study examined associations between demographic, physiological, genetic, and lifestyle-related factors and plasma biomarkers of neurodegeneration. Overall, observed associations were modest, suggesting that blood-based biomarker concentrations may be influenced not only by AD-related pathology but also by individual biological characteristics. These findings highlight the importance of considering potential sources of biomarker variability when interpreting BBBMs in clinical and research settings.

### 4.2 Characteristics of this cohort

The cohort was enriched for individuals at relatively early stages of the AD continuum and characterized by a comparatively low burden of medical comorbidity. This may have contributed to the relatively modest effect sizes observed across several analyses. [49]

### 4.3 Study limitations

While this study provides valuable insights, several limitations should be acknowledged. Firstly, the cohort was characterized by high educational attainment and a low burden of comorbidities, which may limit the generalizability of the findings. The sample size was moderate, comprising 81 individuals with confirmed amyloid positivity and 62 with confirmed negativity, which may have reduced the statistical power of subgroup analyses. Lifestyle factors were primarily evaluated through self-reporting (PASE and PSQI). However, in a subsample of around 50 participants with actigraphy data, the PSQI correlated well with objective measures, thus supporting its value as a marker of perceived sleep quality. Participants were recruited from two trials - the ActiGliA and AmyClear trials, which shared identical inclusion and exclusion criteria but differed in thematic focus. These protocols favored the enrollment of relatively healthy, well-characterized individuals, resulting in a sample that may not fully reflect the demographic and clinical diversity of real-world AD populations. This potential selection bias should be considered when interpreting and generalizing our findings. Finally, the cross-sectional design precludes causal inferences regarding the associations between lifestyle variables and biomarkers.

A substantial proportion of the missing data in this cohort resulted from participants withdrawing or refusing to consent to a lumbar puncture, primarily because the study procedures were perceived as too time-consuming or burdensome. This pattern likely reflects self-selection towards individuals who are more motivated and health-conscious, which could potentially reinforce a healthy cohort bias.

Plasma biomarkers displayed high intrinsic variability, while predictors such as PASE or LDL cholesterol showed limited variance in this health-conscious cohort. Combined with the relatively small sample size and generally small effect sizes, this resulted in wide confidence intervals and limited power to detect weak associations. It is important to note that this reflects statistical uncertainty rather than systematic bias. Furthermore, exact sampling time and fasting status were not available for all participants and may have contributed to additional variability in biomarker measurements. [50] [12] [51] [7].

### 4.4 Strengths of the study

Rigorous biomarker-based selection ensured precise subgroup classification. The multimodal approach, incorporating blood-based, CSF, and imaging markers, provided a comprehensive characterization of AD-related processes. Focusing on modifiable lifestyle factors underscores their potential as intervention targets for AD prevention.

### 4.5 Age and sex effects

In the present analyses, age emerged as one of the most consistent predictors of plasma biomarker concentrations. Higher age was associated with increased plasma NFL concentrations and, to a lesser extent, with higher pTau181 and GFAP levels. In contrast, no consistent age-related associations were observed for the Aβ42/40 ratio. These findings are broadly consistent with previous large-scale studies demonstrating age-related increases in markers of neuroaxonal injury and neurodegeneration, including NFL, GFAP and phosphorylated tau species. [52] [53] Sex-related effects were less consistent. Although previous studies have reported higher NFL concentrations in men and higher GFAP concentrations in women, these associations were not robustly replicated in our cohort. [54] [55] The relatively small sample size and restricted biological variability of this cognitively unimpaired population may have limited statistical power to detect subtle sex-specific effects.

### 4.6 Metabolic and lifestyle factors: cholesterol, creatinine, and physical activity

Markers of renal function represented the strongest non-genetic predictors of plasma biomarker concentrations. In particular, creatinine showed consistent associations with pTau181, NFL and GFAP, supporting previous evidence that circulating neurodegeneration biomarkers are influenced not only by central nervous system pathology but also by peripheral physiological processes, including renal clearance.

[12] [7], [50] [28] These findings reinforce the importance of considering renal function when interpreting blood-based biomarkers in both research and clinical settings. By contrast, associations with LDL cholesterol and physical activity were comparatively modest. Nevertheless, the direction of effects was generally consistent with previous literature linking higher physical activity levels to more favorable biomarker profiles [56] [57] Likewise, altered lipid metabolism remains biologically plausible given its established links to amyloid processing and APOE-related pathways. [3] [6] [7] [12] [13] [50] [51] [58] The relatively high activity levels and restricted variability observed in this healthy cohort may have attenuated detectable effects.

### 4.7 Genetic factors (APOEε4)

As expected, the APOE ε4 genotype was the strongest predictor, explaining most of the variance in plasma ApoE4 levels and significantly lowering the Aβ1–42/1–40 ratio. This is in line with previous reports of stronger ε4-related reductions in the Aβ1–42/1–40 ratio. [59] In contrast, the association between the APOE ε4 genotype and downstream neurodegeneration markers such as NFL and GFAP was less pronounced than in previous reports. [60] [61] [62] While some effects on phosphorylated tau species were observed, these findings should be interpreted with caution due to the smaller sample size available for pTau217 analyses. Overall, the results support previous observations that APOE-related effects are most readily detectable at the level of amyloid biology and may precede measurable changes in markers of neurodegeneration. Consistent with this interpretation, GFAP concentrations were numerically higher in participants with AD but did not differ significantly between diagnostic groups after correction for multiple testing, suggesting that astroglial activation may be less prominent than amyloid-related changes in this relatively early-stage cohort. In any case, the influence of the *APOEε4* genotype has important consequences for interpreting biomarker levels: elevated plasma ApoE4 values make the presence of one or two *APOEε4* alleles highly likely, while the Aβ1–42/1–40 ratio provides complementary information regarding amyloid pathology. Together with phosphorylated tau species, these markers may therefore offer a particularly informative multimarker panel for early Alzheimer's disease detection. [32] This supports the use of multiparametric panels rather than single markers: pTau181 delivers the most consistent signal, while the Aβ1–42/1–40 ratio and ApoE4 provide genotype-dependent, complementary information that helps distinguish between Alzheimer's disease (AD) and healthy controls. In any case, the APOE ε4 genotype exerts pleiotropic effects on both amyloid aggregation and lipid transport, likely due to the ε4 isoform's reduced lipid binding and transport efficiency. [63] [64].

## 5 Conclusion

This study demonstrates that demographic, physiological, genetic, and lifestyle-related factors may contribute to variability in plasma biomarkers of Alzheimer's disease, even within a relatively homogeneous and health-conscious cohort. APOE ε4 status showed the strongest associations, while creatinine, physical activity, sleep quality, and lipid-related measures exhibited more modest relationships with selected biomarkers. These findings highlight the importance of considering potential sources of biological variability when interpreting blood-based biomarkers in clinical and research settings. As blood-based biomarkers move towards broader clinical implementation, understanding the influence of demographic, physiological, and lifestyle-related factors will be important to ensure robust and generalizable application across diverse populations. Future studies should employ longitudinal designs, larger and more diverse cohorts, and standardized assessment procedures to further characterize determinants of biomarker variability and to evaluate their relevance for clinical decision-making. Although blood-based biomarkers show considerable promise for scalable AD detection and monitoring, further validation in real-world populations remains necessary before widespread implementation. [65] [66].

## CRediT authorship contribution statement

**Carolin Kurz:** Writing – review & editing, Writing – original draft, Visualization, Validation, Supervision, Resources, Project administration, Methodology, Investigation, Funding acquisition, Formal analysis, Data curation, Conceptualization. **Marleen Taute:** Writing – review & editing, Writing – original draft, Methodology, Investigation, Data curation. **Paulina Tegethoff:** Writing – review & editing, Writing – original draft, Methodology, Formal analysis, Data curation. **Anna Hufnagel:** Writing – review & editing, Writing – original draft, Methodology, Formal analysis, Data curation, Conceptualization. **Sophia Leonhardt:** Writing – review & editing, Writing – original draft, Investigation, Formal analysis, Data curation, Conceptualization. **Selim Üstün Gürsel:** Writing – review & editing, Writing – original draft, Data curation. **Carolin Koriath:** Writing – review & editing, Writing – original draft, Methodology, Investigation, Formal analysis, Data curation, Conceptualization. **Alexander Jethwa:** Writing – review & editing, Writing – original draft, Methodology, Formal analysis. **Gwendlyn Kollmorgen:** Writing – review & editing, Writing – original draft, Funding acquisition, Formal analysis. **Tobias Bittner:** Writing – review & editing, Writing – original draft, Methodology, Investigation. **Daniel Keeser:** Writing – review & editing, Writing – original draft, Data curation, Conceptualization. **Matthias Brendel:** Writing – review & editing, Writing – original draft, Funding acquisition, Conceptualization. **Jan Haeckert:** Writing – review & editing, Writing – original draft, Investigation. **Julia Utecht:** Writing – review & editing, Writing – original draft, Investigation, Formal analysis. **Boris Papazov:** Writing – review & editing, Writing – original draft, Investigation. **Johannes Levin:** Writing – review & editing, Writing – original draft, Investigation, Funding acquisition, Formal analysis. **Günter Höglinger:** Writing – review & editing, Writing – original draft, Supervision, Project administration, Investigation, Funding acquisition, Formal analysis, Data curation, Conceptualization. **Boris-Stephan Rauchmann:** Writing – review & editing, Writing – original draft, Investigation, Formal analysis, Data curation, Conceptualization. **Robert Perneczky:** Writing – review & editing, Writing – original draft, Supervision, Resources, Project administration, Methodology, Investigation, Funding acquisition, Formal analysis, Data curation, Conceptualization.

## Consent for publication

Not applicable, as no individual person's data are published in this article.

## Ethics approval and consent to participate

The study was approved by the Ethics Committee of LMU Munich (project nos. 17–755, 17–569, 18–606). All participants provided written informed consent in accordance with the Declaration of Helsinki. All participants provided written informed consent prior to participation in the study. The capacity to give consent was assessed in all participants before consent was obtained, in accordance with ethical standards and institutional guidelines.

## Funding

As outlined in the previous publication, this study was supported by the German 10.13039/100005930Research Foundation (DFG) within the framework of the Munich Cluster for Systems Neurology (SyNergy), the Hirnliga (Manfred Strohscheer Foundation) and the 10.13039/501100009318Helmholtz Association and the Pesl-Alzheimer-Foundation. [32] Roche had no role in the interpretation of the data.

## Declaration of competing interest

The authors declare no competing interests relevant to this study. Some authors are affiliated with Roche; however, the company had no role in study design, data collection, analysis, interpretation, manuscript preparation, or the decision to submit. Roche Diagnostics supported plasma biomarker measurements. Author disclosures related to Roche and other companies have been reported previously. [32]

## Data Availability

Access to the data supporting this study's findings is restricted to protect participant privacy but may be granted upon reasonable request to the corresponding author.
