# Supplementary material for: Health-related factors and their impact on blood-based biomarkers of Alzheimer's disease
Source: Aging Brain. 2026 Sep 3;10:100166. doi: 10.1016/j.nbas.2026.100166 (PMC13571739; doi:10.1016/j.nbas.2026.100166)
Supplement: Supplementary file 1 [file mmc1.docx]

**Health-Related Factors and Their Impact on Blood-Based Biomarkers of Alzheimer’s Disease**

**This Supplement contains:**

Figure S1: Sleep-activity questionnaires in relation to actigraphy

Figure S2: Scree plot of principal component analysis (PCA) for actigraphy-derived sleep parameters

Table S1: Robust Regression Analyses of Actigraphy Measures and Plasma/CSF Biomarkers in CSF-Confirmed Baseline Participants

Table S2: Descriptive Statistics for Smoking and Alcohol Intake

**Figure S1: Sleep-activity questionnaires in relation to actigraphy**

Legend Figure S1:   Partial correlations between actigraphy-derived sleep parameters and self-report questionnaires. Color intensity indicates the strength and direction of the correlation (blue = positive, red = negative). Values represent partial correlation coefficients (r); *p < .05, **p < .01. Questionnaire availability in the total sample (N = 180): ESS n = 135 (75.0%), PASE n = 109 (60.6%), PSQI n = 119 (66.1%), RBDSQ n = 121 (67.2%); complete questionnaire set (all four scales) n = 90 (50.0%). Questionnaires: RBD-SQ = REM Sleep Behavior Disorder Screening Questionnaire; PSQI = Pittsburgh Sleep Quality Index; PASE = Physical Activity Scale for the Elderly (semi-quantitative self-report; higher scores indicate greater physical activity); ESS = Epworth Sleepiness Scale. Actigraphy variables: Awakenings (n) = number of nocturnal awakenings; Sleep Efficiency (%) = ratio of total sleep time to time in bed; Time in Bed (h) = total time spent in bed; Total Sleep Time (h) = objectively measured sleep duration; WASO (min) = Wake After Sleep Onset.

**Figure S2: Scree plot of principal component analysis (PCA) for actigraphy-derived sleep parameters**

**Legend Figure S2:** The Kaiser–Meyer–Olkin measure of sampling adequacy was 0.47, and Bartlett’s test of sphericity confirmed suitability for factor analysis (χ²(55) = 562.91, p < 0.001). According to the scree plot and the Kaiser criterion, four components were retained, together explaining 79.2% of the total variance (RC1: 23.9%, RC2: 22.3%, RC3: 18.1%, RC4: 14.9%). RC1 captured overall sleep duration (e.g., total sleep time, time in bed), RC2 reflected nocturnal sleep continuity (positive loadings for WASO and awakenings, negative loading for SEff), RC3 described daytime activity and rhythm stability (M10, IS), and RC4 was dominated by high RA values and low L5 counts, indicating circadian amplitude. For subsequent analyses, SEff (from RC2) and RA (from RC4) were selected as representative actigraphy markers.

**Table S1: Associations between actigraphy measures and plasma and CSF biomarkers in CSF-confirmed baseline participants**

| **Outcome** | **Predictor** | **β** | **95% CI** | **p raw** | **p FDR** | **n** |
| --- | --- | --- | --- | --- | --- | --- |
| CSF pTau181  (log) | RA | 0.99 | 0.22 to 1.76 | 0.017 | 0.089 | 43 |
| Plasma Aβ42/40 ratio  (log) | RA | 0.17 | −0.04 to 0.37 | 0.116 | 0.381 | 39 |
| CSF total tau  (log) | RA | 1.27 | −0.46 to 3.01 | 0.157 | 0.409 | 43 |
| Plasma pTau217  (log) | SE | 0.023 | −0.011 to 0.057 | 0.199 | 0.409 | 42 |
| CSF pTau181  (log) | SE | −0.014 | −0.036 to 0.007 | 0.205 | 0.409 | 43 |
| Plasma pTau181  (log) | RA | 0.34 | −0.21 to 0.89 | 0.237 | 0.445 | 37 |
| CSF total tau  (log) | SE | −0.020 | −0.057 to 0.017 | 0.295 | 0.502 | 43 |
| Plasma Aβ42/40 ratio  (log) | SE | −0.005 | −0.013 to 0.004 | 0.298 | 0.502 | 39 |

Supplementary Table S1. Robust regression analyses examining associations between sleep efficiency (SE) and relative amplitude (RA; (M10 − L5)/(M10 + L5)) with plasma and CSF biomarkers in CSF-confirmed baseline participants. Models were adjusted for age and sex. P values were FDR-corrected. No association remained significant after correction

**Table S2: Descriptive Statistics for Smoking and Alcohol Intake**

| **Variable** | **Mean SD AD** | **Mean SD HC** | **n AD** | **n HC** | **p** |
| --- | --- | --- | --- | --- | --- |
| Cigarettes per Day | 0.6 (2.8) | 0.33 (1.17) | 75 | 41 | 0.581 |
| Alcohol Units per Day | 1.12 (0.98) | 1.5 (1.06) | 75 | 41 | 0.152 |

Legend Table S2: An “alcohol unit” in the UK equals 10 ml (8 g) of pure alcohol, roughly half a pint of beer (4% Alcohol by Volume - ABV), a small glass of wine (12% ABV), or a single shot of spirits (40% ABV).
